# Innocent Tumours of the Rectum

**Published:** 1894-08-11

**Authors:** Alexander Miles

**Affiliations:** Surgical Tutor Royal Infirmary, Edinburgh


### Medical Progress and Hospital Clinics.

[*The Editor will be glad to receive offers of co-operation and contributions from members of the profession. All letters should be addressed to* The Editor, The Lodge, Porchester Square, London, W.]

### INNOCENT TUMOURS OF THE RECTUM.

By Alexander Miles, M.D., F.R.C.S.Edin., Surgical Tutor Royal Infirmary, Edinburgh.

The relative frequency of non-malignant tumours of the rectum seems to vary very considerably in the experience of different writers on this subject. Some authorities appear to look upon them as comparatively rare, while others include them among the commoner affections of this part. Considerable confusion also exists as to their nomenclature, principally on account of the loose and unscientific way in which the term "polypus" is used in connection with them. The word is an old and convenient one, and so long as it is qualified by another, indicating the histological structure of the tumour, may with advantage be retained. Used alone, however, it covers much pathological ignorance, as it is made to include practically all the benign tumours of the rectum.

**Varieties.**—The varieties of innocent tumour met with in the rectum are (1) adenoid polypus (adenoma), (2) fibrous polypus (fibroma), (3) papillomatous polypus (papilloma), (4) cystic tumour (cystoma), (5) dermoid tumour (teratoma), (6) fatty tumour (lipoma), (7) "polypoid growths," (8) warty growths. Of these the first three are by far the commonest, the others being more or less of the nature of pathological curiosities, and calling for little remark here.

It is believed by some[1] that the different varieties of polypus—adenoid, fibrous, and papillomatous—are to be looked upon as essentially the same in nature and mode of origin, and that they only differ inasmuch as the relative amount of their constituent tissues is variable. Others, again, and among them Mr. Harrison Cripps,[2] considers the difference one of kind rather than of degree.

I. **Adenoid Polypus** (Adenoma. Mucous polypus).[6]—This, by far the commonest form of innocent rectal tumour, consists in a great hypertrophy of the follicles of Lieberkuhn, which abound in the mucous membrane, or of the mucous follicles, or of both. Supporting these is a quantity of areolar and fibrous tissue, in which run small blood vessels for the supply of the growth. The columnar epithelium which covers the tumour is directly continuous with that of the mucous membrane of the intestinal wall. Structurally, therefore, this form of tumour is, as Mr. Cripps puts it, "an extreme exaggeration of the plan upon which the mucous membrane of the rectum is constructed." As the mass increases it stretches the tissue which connects it to the bowel wall, and a pedicle is formed, which often attains considerable length, averaging from two to three inches.

Most authorities agree that the adenoma is much commoner in children below the age of ten than in adults, but Mr. Allingham's[3] statistics lead him to believe that there is no great disproportion as regards the age of the sufferers. Allowance has to be made, however, as he remarks, for the undoubted fact that many polypi are spontaneously shed by children, the weak pedicle giving way during straining.

The point of attachment is most frequently on the dorsal aspect of the rectal wall, about one and a half to two inches from the anus. Seldom is it much nearer the opening, and not unfrequently it is further off. Usually, but not always, the tumour is solitary, and varies in size from a bean to a hen's egg. Observers agree in comparing the growths to a raspberry or half-ripe mulberry as to size, shape, and colour.

*Symptoms and Diagnosis.*—Many adenomata give rise to no symptoms during life, and their presence is only detected on post-mortem examination. In most cases, however, they are more troublesome. The child has a frequent desire to go to stool, and the action of the bowels is accompanied by much tenesmus and the escape of a considerable quantity of mucus. Bleeding during and after defecation is a very common accompaniment, and the association of this with long-continued irritation in the rectum is a valuable diagnostic guide. When the pedicle is sufficiently long to permit of it, the tumour is extruded from the anus, and is returned with considerable pain. The diagnosis is not difficult. The age of the patient renders piles unlikely. A digital examination should be made after the bowels have been emptied by an enema, and the finger passed as far into the gut as possible, and then, as it is being withdrawn, swept round the rectal wall in order to detect the site, size, and length of the pedicle. The use of the speculum will remove any doubt which may remain.

In children the condition is rarely complicated, but it may be the cause of an intussusception or of prolapse of the rectum.

*Treatment.*—This is exceedingly simple. The bowel is emptied by an enema, the tumour brought down to the anus, the pedicle transfixed with a double silk ligature, and tied in two halves as close up to the mucous membrane as possible. The polypus is then cut off with scissors. A narrow pedicle may be tied in one ligature. The bowels are kept at rest for a day or two, and subsequently opened by enemata.

II. **Fibrous Polypus** (Fibroma).—This tumour is much rarer than the adenoma. It begins as an hypertrophy of a small portion of the sub-mucous tissue of the bowel, which gradually increases in size, and is covered by the normal mucous membrane. It is lobulated and smooth on the surface, and on section is pale and firm, cutting with a creak not unlike that of a scirrhus tumour of the breast. It may attain to a considerable size, as large as a mandarin orange, or even larger.[4] The pedicle is as a rule shorter and broader than that of an adenoma, and is always attached above the sphincters. This variety is almost invariably found in adults.

*Symptoms.*—It causes little or no inconvenience so long as the pedicle remains too short to allow it to be protruded during defecation. A common history is that one day, without any previous discomfort, when at stool, patient became aware of a mass protruding from the rectum, because of the severe pain it occasioned. This is accompanied by a loss of blood varying in amount. Much pain in the lumbar region is sometimes complained of. Each time the bowels move the tumour is nipped by the sphincters, and the pain and bleeding recur. The writer recently met with a case in which a long finger-like lobule depended from the lowest part of the tumour, and was firmly grasped by the internal sphincter, giving rise to continuous pain and tenesmus with watery diarrhœa, and causing gangrene of the part nipped. On removal a distinct groove round the lobule indicated where it had been grasped.

Fibrous polypus is often associated with ulcer and fissures of the rectum, these forming where the tumour lies against the mucous membrane. Abscess also is not unknown as a sequel.

*Treatment.*—The treatment is carried out on the same lines as for the simple adenoid polypus. The pedicle, being larger and more vascular, will require more attention to prevent hæmorrhage, for which the double ligature is quite sufficient. Subsequent enemata of boracic acid lotion are advantageous.

III. **Papillomatous Polypus** (Papilloma. Vellous tumour).—Still rarer than the fibroma is the papilloma recti. Mathews[5] considers it the rarest of all rectal tumours, but this does not seem to be the case. It is composed of branched outgrowths from the mucous membrane, consisting of a basis of connective tissue, carrying a small artery and vein and covered by columnar epithelium. From the main papillæ secondary ones grow, and from these others still smaller. They are met with in patients usually over forty years of age, and are said to occur in different members of the same family[1]. They have a short broad pedicle, or are actually sessile, and to the feel are characteristically soft and velvety, and slightly lobulated.

*Symptoms.*—The most characteristic clinical feature of this variety is the large exudation of glairy sticky mucous discharge to which it gives rise; but bleeding is also a prominent symptom. Pain, on the other hand, is neither constant nor severe. Protrusion at stool is not usual.

*Treatment.*—As these tumours not unfrequently recur after removal, and are therefore by some believed to lie on the borderland between the innocent and the malignant, any attempt at removal must be complete. The sphincter is dilated with the patient anæsthetised. The growth is seized with vulsellum forceps, its broad base transfixed with strong silk, and tied so as to embrace only healthy tissues. The tumour is then completely cut away. Mr. Allingham says that when they grow from the anterior rectal wall (an unusual situation), it is possible for a pouch of peritoneum to be pulled down into the pedicle. It does not seem a probable contingency.

**IV., V., VI. Cystic, Dermoid, and Fatty Tumours** of the rectum are exceedingly rare, and the detailed consideration of them would be out of place here. The cysts as a rule are serous; the dermoid tumour has the ordinary contents of such growths, hair, teeth, bone, &c.; and the lipoma has the usual features of its kind. They are all treated by removal.

**VII. Polypoid Growths.**—Mr. Allingham describes, under this term, a form of enlargement which consists in a number of small tags of mucous membrane. They are usully placed near a pile or fissure, and are of importance because they give rise to a profuse watery discharge which is apt to aggrevate, if it does not actually cause a fissure, or to induce pruritis ani. They are to be snipped off with scissors.

**VIII. Warty Growths**, strictly speaking, are not tumours of the rectum—although they sometimes do occur in the bowel—but grow round the anus. They are treated like warts elsewhere by excision, or by the application of nitric acid with subsequent scraping.

[1] Dict. Surg. Art. Dis. Rectum: [2] Diseases of Rectum. [3] Diseases of Rectum, 5th ed. [4] Syme, Contributions to Pathology and Practice of Surgery, 1848. [5] Diseases of Rectum, Anus, and Sigmoid Flexure, 1892. [6] Miller, Edin. Med. Jour., 1870.

